# Genetic predisposition to longer telomere length and risk of childhood, adolescent and adult-onset ependymoma

**DOI:** 10.1186/s40478-020-01038-w

**Published:** 2020-10-28

**Authors:** Chenan Zhang, Quinn T. Ostrom, Eleanor C. Semmes, Vijay Ramaswamy, Helen M. Hansen, Libby Morimoto, Adam J. de Smith, Melike Pekmezci, Zalman Vaksman, Hakon Hakonarson, Sharon J. Diskin, Catherine Metayer, The Glioma International Case-Control Study (GICC), Elizabeth B. Claus, Dora Il’yasova, Kyle M. Walsh, Joellen Schildkraut, Jill S. Barnholtz-Sloan, Sara H. Olson, Jonine L. Bernstein, Christoffer Johansen, Robert B. Jenkins, Beatrice S. Melin, Margaret R. Wrensch, Richard S. Houlston, Melissa L. Bondy, Michael D. Taylor, Joseph L. Wiemels, Melissa L. Bondy, Kyle M. Walsh

**Affiliations:** 1 Department of Epidemiology and Biostatistics, University of California, San Francisco, San Francisco, USA; 2 Department of Medicine, Section of Epidemiology and Population Sciences, Dan L. Duncan Comprehensive Cancer Center, Baylor College of Medicine, Houston, USA; 3 Medical Scientist Training Program, Duke University School of Medicine, Durham, USA; 4 Children’s Health and Discovery Initiative, Department of Pediatrics, Duke University, Durham, USA; 5 The Arthur and Sonia Labatt Brain Tumor Research Centre, The Hospital for Sick Children, Toronto, Canada; 6 Department of Neurological Surgery, University of California, San Francisco, San Francisco, USA; 7 School of Public Health, University of California, Berkeley, Berkeley, USA; 8 Center for Genetic Epidemiology, University of Southern California, Los Angeles, USA; 9 Department of Pathology, University of California, San Francisco, San Francisco, USA; 10 Department of Biomedical and Health Informatics, Children’s Hospital of Philadelphia, Philadelphia, PA USA; 11 Center for Applied Genomics, Children’s Hospital of Philadelphia, Philadelphia, PA USA; 12 Department of Pediatrics, Children’s Hospital of Philadelphia and Perelman School of Medicine, University of Pennsylvania, Philadelphia, USA; 13 Department of Epidemiology and Population Health, Stanford University, Palo Alto, CA USA; 14 Department of Neurosurgery and Duke Cancer Institute, Duke University School of Medicine, DUMC Box 3050, Durham, NC 27710 USA

**Keywords:** Ependymoma, Pediatric cancer, Telomere length, Mendelian randomization

## Abstract

Ependymoma is the third most common brain tumor in children, with well-described molecular characterization but poorly understood underlying germline risk factors. To investigate whether genetic predisposition to longer telomere length influences ependymoma risk, we utilized case–control data from three studies: a population-based pediatric and adolescent ependymoma case–control sample from California (153 cases, 696 controls), a hospital-based pediatric posterior fossa type A (EPN-PF-A) ependymoma case–control study from Toronto’s Hospital for Sick Children and the Children’s Hospital of Philadelphia (83 cases, 332 controls), and a multicenter adult-onset ependymoma case–control dataset nested within the Glioma International Case-Control Consortium (GICC) (103 cases, 3287 controls). In the California case–control sample, a polygenic score for longer telomere length was significantly associated with increased risk of ependymoma diagnosed at ages 12–19 (P = 4.0 × 10^−3^), but not with ependymoma in children under 12 years of age (P = 0.94). Mendelian randomization supported this observation, identifying a significant association between genetic predisposition to longer telomere length and increased risk of adolescent-onset ependymoma (OR_PRS_ = 1.67; 95% CI 1.18–2.37; P = 3.97 × 10^−3^) and adult-onset ependymoma (P_MR-Egger_ = 0.042), but not with risk of ependymoma diagnosed before age 12 (OR = 1.12; 95% CI 0.94–1.34; P = 0.21), nor with EPN-PF-A (P_MR-Egger_ = 0.59). These findings complement emerging literature suggesting that augmented telomere maintenance is important in ependymoma pathogenesis and progression, and that longer telomere length is a risk factor for diverse nervous system malignancies.

## Introduction

Ependymoma is the third most common brain tumor in children, accounting for 5–10% of childhood brain tumors, with more than half of all cases occurring in children under five years old. Most pediatric ependymomas are intracranial in origin (90%), whereas a greater proportion of adult-onset ependymomas occur in the spinal cord (66%) [40]. The molecular characterization of ependymal tumors is well-described and may inform a new era of precision diagnostics and targeted therapies [25, 41]. Underlying germline risk factors that predispose individuals to develop ependymoma remain poorly understood, as the ability to perform genetic epidemiology studies of rare diseases is limited and traditional genome-wide association study (GWAS) approaches are underpowered. However, alternative analytic approaches that use polygenic scores or Mendelian randomization analyses to model genetic predisposition to “intermediate phenotypes” hold promise for advancing our understanding of genetic risk in rare diseases, including childhood cancers [6, 48]. Telomere length is perhaps the “intermediate phenotype” that has been best-characterized for its association with brain tumor risk, as genetic predisposition to longer telomeres increases risk of both adult glioma [52, 53] and meningioma [36]. Despite the known association between genetic predisposition to longer telomere length and certain adult-onset brain tumors, the association between ependymoma risk and telomere length has not been evaluated in either children or adults.

Telomeres are nucleoprotein structures that protect the ends of chromosomes during normal cellular DNA replication; however, telomeres shorten with each replicative cell division cycle until reaching a critically short length, at which point cellular senescence or apoptosis ensues [5, 16]. Telomere length is maintained during cellular replication by the enzyme telomerase, encoded by the *TERT* gene. Normally, telomerase is active in stem and progenitor cells, yet activity is repressed in normal somatic cells as an anti-proliferative mechanism [15]. An important hallmark of cancer is “enabling replicative immortality,” which is necessary for sustained malignant growth and which is often achieved by reactivating telomerase expression in immortalized cells [26]. Cancer cells are able to avoid senescence and apoptosis in part by maintaining telomere length indefinitely. This is typically achieved through telomerase reactivation or through a homologous recombination-associated process referred to as alternative lengthening of telomeres (ALT) [26, 37]. While dysregulated telomere biology has been implicated in ependymoma progression and prognosis [46, 50, 51], the *TERT* promoter mutations associated with telomerase reactivation are uncommon in both childhood and adult ependymomas, as are the *ATRX* mutations associated with ALT [9]. Individuals who are genetically predisposed to longer telomere length or more efficient telomere maintenance are at increased risk of adult glioma and childhood neuroblastoma [53, 55], perhaps due to an enhanced capacity for pre-malignant cells to divide and acquire additional oncogenic mutations before their telomere reserves are depleted [2, 56]. However, it is unknown whether genetic predisposition to telomere length contributes to ependymoma risk.

To investigate whether genetic predisposition to telomere length influences ependymoma risk, we examined the association between validated genetic instruments associated with longer leukocyte telomere length (LTL) and ependymoma risk in case–control analyses [14]. We utilized both polygenic scores modeling genetic predisposition to longer LTL and Mendelian randomization analyses to test for a causal association between longer LTL and ependymoma risk. Because a previously observed association between polygenic scores for telomere length and neuroblastoma risk implicated effect modification by age [55], we also performed age-stratified analyses in groupings defined a priori. The current study utilizes case–control data from three different collaborations, including: (1) a population-based pediatric and adolescent ependymoma case–control sample from California, (2) a hospital-based pediatric posterior fossa type A (PFA) ependymoma case–control study from Toronto’s Hospital for Sick Children and the Children’s Hospital of Philadelphia, and (3) a multicenter adult-onset ependymoma case–control dataset nested within the Glioma International Case-Control Consortium (GICC).

## Methods

### Ethics statement

The study was approved by Institutional Review Boards at The University of California, Berkeley, The University of California, San Francisco, the California Department of Public Health, The Children’s Hospital of Philadelphia, the University of Toronto Hospital for Sick Children, and Baylor College of Medicine.

### California Cancer Record Linkage Project (CCRLP) case–control dataset

Blood samples from neonates born within the state of California are collected by the California Department of Public Health, Genetic Diseases Screening Branch for the purpose of disease screening, with remaining samples archived at − 20 °C since 1982 and made available for approved research. We linked statewide birth records from the California Department of Public Health for the years 1982–2009 to data from the California Cancer Registry (CCR) for diagnosis years 1988–2011. Cases were defined as patients diagnosed with ependymoma before age 20, per CCR record of 2014 ICD-O-3 codes 9391-9394. Controls were matched on race/ethnicity, sex, month and year of birth from the pool of children born in California during the same period and not reported to CCR as having any childhood cancer. Included in this analysis were 153 non-Hispanic white children with ependymoma and 696 controls, as previously described [62]. Subjects from other racial/ethnic backgrounds were not included due to the questionable performance of polygenic scores built with loci and effect estimates derived from European-ancestry populations and applied to Hispanic or African-American populations [24, 33], and further the lack of suitable GWAS of telomere length in these populations [10]. Details on the linkage and use of neonatal bloodspots for studying pediatric cancers have been reported previously [57].

### CCRLP DNA extraction and genotyping

Details on the use of neonatal bloodspots for DNA extraction and genotyping have been reported previously [61]. In brief, DNA was extracted from a one-third portion of a 12-mm dried blood spot using the QIAamp DNA Investigator Kit (Qiagen), followed by addition of 280 μL of Buffer ATL and 20 μL of Proteinase K to each sample. Samples were vortexed and then incubated in a dry-bath shaker at 900 rpm and 56 °C for one hour. Samples were then briefly centrifuged, after which the lysate solution was transferred to a new 2 mL microcentrifuge tube, and the solid remnants discarded. 1 μL of 1 ng/μL carrier RNA was added to the lysate, briefly vortexed, and placed in the Qiagen Qiacube automated work station for DNA isolation, yielding a purified DNA sample in ATE buffer. DNA was genotyped on the Affymetrix Axiom World Array (LAT), with average genotype concordance > 99% between duplicate samples on the same plates. Quality-control procedures included call-rate filtering for single nucleotide polymorphisms (SNPs) and samples, performed iteratively by removing SNPs with call rates < 92%, then samples with call rates < 95%, then SNPs with call rates < 97%, then samples with call rates < 96%. SNPs with significant departure from Hardy–Weinberg equilibrium (P < 1.0 × 10^−5^) among controls were excluded. Samples with mismatched reported versus genotyped sex were also excluded. Identity-by-descent (IBD) analyses were performed in PLINK on cases and controls, with exclusion of one member of any sample pair that had an identity-by-descent proportion > 0.18 [44]. Using SNP array data from the HapMap phase III European reference panel samples, we calculated ancestry-informative principal components (PCs) and removed any sample showing evidence of non-European ancestry (> 3 SDs from mean CEPH values on PCs 1–3). We performed haplotype phasing and imputation with SHAPEIT v2.79029 and Minimac3 software using phased genotype data from the 2016 release of the Haplotype Reference Consortium [17, 34]. SNPs with imputation quality (INFO) scores < 0.60 or posterior probabilities < 0.90 were excluded.

### Toronto case-control dataset

A total of 83 pediatric ependymoma patients (median age 3 years) and 332 control children of non-Hispanic white ethnicity were genotyped on the Illumina OmniExpress array at the Center for Applied Genomics (CAG) at the Children’s Hospital of Philadelphia (CHOP). All ependymoma patients were recruited onto study at The Hospital for Sick Children at The University of Toronto and had posterior fossa type A (PF-EPN-A) tumors, as determined by integrated analysis of DNA methylation, copy-number, gene expression, and clinical parameters, as previously described [41]. DNA was extracted from blood where available, but a subset of patient DNA specimens were extracted from tumor specimen to increase sample size. Because PF-EPN-A are genetically bland and rarely harbor either point mutations or copy-number alterations, including in the regions of chromosomes 2, 3, 4, 5, 10, 17, 19, and 20 where SNPs used as genetic instruments for telomere length are located, deviation from constitutive genotypes appeared minimal [41]. GWAS data underwent quality-control procedures as previously described [19, 32]. Genotypes were phased using SHAPEIT2 [18] and whole-genome imputation was performed using IMPUTE2 [29] with 1000 Genomes Phase 3 release as the imputation reference panel [22]. Case–control comparisons were performed using the frequentist test with an additive model and score method to deal with uncertainty as implemented in SNPTEST v2.4.1 [32], with adjustment for the ten five ancestry-informative PCs.

### Glioma International Case-Control dataset (GICC)

GICC is the largest glioma study to-date including biospecimens and blood samples, conducted by the Genetic Epidemiology of Glioma International Case-Control Consortium [1]. Individual-level genotype and phenotype data are available for download from the Database of Genotypes and Phenotypes (dbGaP, Study Accession phs001319.v1.p1) after review and approval by the NCI Data Access Committee. From the GICC data, a subset of 103 adult ependymoma cases age 18–72 (with three cases < age 20) and 3287 controls were selected. Subject recruitment and control selection has previously been described in detail [1].

### Single SNP and polygenic score analyses

We investigated the individual effect of eight telomere-length associated SNPs on ependymoma risk in the CCRLP, Toronto, and GICC datasets. We also assessed the combined effect of these SNPs in CCRLP cases and controls, where individual-level genotype data were available (for Toronto and GICC subjects, only SNP-level summary statistics were available). SNPs were chosen based on strong prior evidence of association with LTL in previous GWAS publications demonstrating genome-wide significant associations (P < 5 × 10^−8^) and excluding linked SNPs (R^2^ < 0.05) in order to avoid “double-counting” risk loci [14]. Although the proportion of variation explained by the eight SNPs is individually small (~ 2%) [14], the genotypically-estimated relative LTL across individuals ranged from 140 to 943 base pairs. This 803 base-pair range corresponds to > 25 years of age-related telomere attrition (based on an average LTL attrition rate of 20–40 base pairs/year) [21]. We first assessed single LTL SNP associations with ependymoma risk using logistic regression, assuming an allelic additive model for 0, 1 or 2 copies of the allele for longer LTL. For CCRLP subjects, we also constructed polygenic scores for longer LTL by calculating the weighted sum of the number of alleles corresponding to longer LTL (up to 16 alleles from 8 unlinked SNPs) for each individual, in which the weight was taken as the effect estimate from the LTL GWAS from the ENGAGE Consortium Telomere Group [14]. We performed a logistic regression analysis of the standardized polygenic scores for longer LTL and ependymoma risk, adjusting for sex and the top 10 principal components. Resulting beta estimates are interpreted as the difference in ependymoma risk per one standard deviation increase in the LTL score. We also assessed the differences in LTL and ependymoma association stratified by age (< 12 years old vs. ≥ 12 years old) and tumor location (spinal vs. intracranial) in CCRLP data. We used PLINK to complete both the single SNP and polygenic score association analyses.

### Mendelian randomization analyses

Mendelian randomization (MR) is a causal inference method in which genetic variants are used as instrumental variables, *i.e.* proxies for a risk factor of interest, to evaluate the causal relationship between the risk factor and an outcome of interest. In the two-sample summary data MR approach, summary statistics for SNP-exposure associations are obtained from a different set of samples from those for the SNP-outcome association, assuming both samples are drawn from the same underlying population [11]. The polygenic score association analysis can be considered a form of MR analysis in which the score is considered an instrumental variable [12], assuming that all variants contributing to the score are valid instruments that do not violate any of the three MR assumptions. However, a formal application of the MR method, along with various sensitivity analyses, are necessary as prior studies show that risk score association analyses can suffer from higher false positive rates due to horizontal pleiotropy, a violation of the MR assumptions [28, 45]. Furthermore, in the absence of individual-level data SNP data, the two-sample summary data MR method can be useful for approximating the association between a genetic score and an outcome of interest using summary statistics. Our MR analyses used summary statistics for the association with ependymoma risk of the same 8 SNPs used in our polygenic score model, adjusted for sex and 10 principal components. We used the inverse-variance weighted (IVW) method, along with the (1) MR-Egger method, which provides consistent estimates in the presence of horizontal pleiotropy given that pleiotropic effects are independent of instrument strength across all variants; [7], (2) the weighted median method [8], which provides consistent estimates even when up to 50% of the information comes from invalid instruments; and (3) the mode-based method [27], which provides consistent estimates even when a majority of instruments are invalid, to assess the causal association between LTL and ependymoma risk in CCRLP data, Toronto data, and GICC data. We used the MendelianRandomization R package for these analyses [59].

## Results

From the CCRLP dataset, a total of 153 non-Hispanic white pediatric ependymoma patients and 696 controls were available for analyses after linkage, newborn bloodspot DNA extraction, genotyping, QC procedures, and SNP imputation. Demographics and clinical features of the CCRLP ependymoma cases are shown in Additional file 1: Table S1. Demographic details on the Toronto (n = 83 cases, 332 controls) and the GICC (n = 103 cases, 3287 controls) case–control datasets have been previously described [1, 62].

A total of eight SNPs previously associated with LTL at genome-wide significant levels and independently replicated by Codd et al. [14] were successfully genotyped in all three ependymoma datasets: rs11125529 (*ACYP2*), rs10936599 (*TERC*), rs7675998 (*NAF1*), rs2736100 (*TERT*), rs9420907 (*OBFC1*), rs3027234 (*CTC1*), rs8105767 (*ZNF208*), and rs755017 (*RTEL1*). Two nominally significant associations (P_unadjusted_ < 0.05) were observed in the CCRLP age-stratified analysis for patients ≥ 12 years old at diagnosis at rs10936599 in *TERC* (OR = 1.98; 95% CI = 1.06, 4.06; P = 0.043) and at rs7675998 in *NAF1* (OR = 2.15; 95% CI = 1.09, 4.74; P = 0.039), but no associations were observed for CCRLP patients < 12 (Additional file 1: Table S2). One nominally significant association (P < 0.05) was observed in the Toronto PF-A case–control sample at rs9420907 in *OBFC1* (OR = 1.71; 95% CI = 1.07, 2.73; P = 0.026), but no single-SNP associations were observed in the GICC adult ependymoma data (Additional file 1: Table S3).

The association between a polygenic score for longer LTL and pediatric ependymoma risk was assessed using logistic regression, adjusting for sex and 10 PCs. Among all California cases and controls age ≤ 19, a non-significant association was observed (OR = 1.12; 95% CI 0.94–1.34; P = 0.207, Table 1). When cases were stratified by age at diagnosis, using cutoffs defined a priori based on previous observations in neuroblastoma [55], a significant association was observed between longer LTL score and increased risk of ependymoma in CCRLP patients diagnosed at ≥ 12 years of age (OR = 1.67; 95% CI 1.18–2.37; P = 3.97 × 10^−3^). However, the LTL score was not associated with ependymoma risk in patients < 12 years old (OR = 1.12; 95% CI 0.94–1.34; P = 0.21). In a case–case comparison to test for etiologic heterogeneity by age at diagnosis, the LTL score was significantly higher in adolescent-onset ependymoma patients (12–19 years of age) compared with childhood-onset ependymoma patients (0–12 years of age) (P = 0.021). When further stratified by tumor location, longer LTL was associated with increased risk of both intracranial and spinal ependymoma diagnosed at ≥ 12 years of age (P = 0.048 and 0.024, respectively). However, longer LTL was not associated with increased risk of intracranial ependymoma in children diagnosed before age 12 (P = 0.53), and longer LTL was inversely associated with risk of spinal ependymoma in children under 12 (P = 8.9 × 10^−3^) (Additional file 1: Table S4). When intracranial ependymoma were further restricted to supratentorial tumors, longer LTL was again significantly associated with increased risk in those diagnosed at ≥ 12 years of age (P = 0.024), but no among those diagnosed before 12 years of age (P = 0.86) (Additional file 1: Table S4).

**Table 1:** Association between a polygenic score for longer telomere length and risk of ependymoma in the California Cancer Record Linkage Project case–control dataset (ages 0–19)

Logistic regression model	Cases/controls	OR (95% CI)^a^	P-value	
All patients combined	153/696	1.12 (0.94–1.34)	0.207	
Childhood-onset^c^	114/696	0.99 (0.81–1.21)	0.939	P_case-case_ = **0.021**^**b**^
Adolescent-onset^d^	39/696	1.67 (1.18–2.37)	**3.97** × 10^**−3**^	

Nominally significant P values < 0.05 in bold

^a^Adjusted for sex and 10 principal components

^b^P-value corresponding to the association between a polygenic score for longer telomere length and age at diagnosis (< 12 vs. ≥ 12) in a case-only analysis of ependymoma patients

^c^less than 12 years of age at diagnosis

^d^12–19 years of age at diagnosis

Formal Mendelian randomization analyses were conducted to make causal inferences about the association between telomere length and ependymoma risk in all three datasets. The MR results complemented the polygenic score analyses in CCRLP data, with IVW MR estimates suggesting a significant causal association between longer LTL and increased ependymoma risk in patients diagnosed at ≥ 12 years of age (P_IVW_ = 6.3 × 10^−3^). Sensitivity analyses supported this link, finding no evidence of directional pleiotropy with MR Egger tests (P_MR-intercept_ = 0.67). A similar positive association was observed in GICC adult ependymoma data using the MR Egger estimate (P_MR-Egger_ = 0.042), where a non-zero intercept term suggested the presence of directional pleiotropy (P_MR-intercept_ = 0.059). Weighted-median and mode-based MR analyses also show positive associations in CCRLP adolescent (age ≥ 12) and in GICC adult ependymoma, although effect sizes were smaller and several confidence intervals included the null (Table 2).

**Table 2:** Mendelian randomization analysis of the association between longer LTL score and ependymoma risk in CCRLP, Toronto, and GICC case–control datasets

MR Estimate	Pediatric EPN-PF-A (Toronto)^a^		Childhood-onset^b^ (CCRLP)		Adolescent-onset^c^ (CCRLP)		Adult-onset^d^ (GICC)	
	OR (95% CI)	P-value	OR (95% CI)	P-value	OR (95% CI)	P-value	OR (95% CI)	P-value
IVW	0.51 (0.03, 8.7)	0.64	0.93 (0.15, 5.7)	0.94	91.7 (3.6, 2.3 × 10^3^)	**0.0063**	2.01 (0.34, 12.0)	0.43
Weighted median	0.61 (0.03, 13.3)	0.76	0.82 (0.08, 7.9)	0.85	368 (4.1, 3.3 × 10^4^)	**0.010**	2.82 (0.27, 29.0)	0.38
Mode-based	0.67 (0.02, 22.5)	0.82	0.30 (0.02, 4.9)	0.40	690 (0.42, 1.1 × 10^6^)	0.084	4.05 (0.30, 4.01)	0.30
MR Egger	183 (1.0 × 10^−6^, 3.3 × 10^10^)	0.59	0.08 (2.8 × 10^−4^, 23.1)	0.38	793 (0.02, 3.2 × 10^7^)	0.22	294 (1.23, 7.0 × 10^4^)	0.042
(Intercept)	0.61 (0.13, 2.94)	0.45	1.19 (0.81, 1.8)	0.37	0.86 (0.43, 1.7)	0.67	0.70 (0.48, 1.01)	0.059

Adjusted for subject sex and 10 ancestry-informative principal components

Nominally significant P values < 0.05 in bold

^a^Sample size for Toronto posterior fossa type A pediatric ependymoma case–control study includes 83 cases and 332 controls

^b^Sample size for CCRLP childhood-onset case–control subset includes 114 cases (age < 12 years) and 696 controls

^c^Sample size for CCRLP adolescent-onset case–control subset includes 39 cases (age 12–19 years) and 696 controls

^d^Sample size for Glioma International Case-Control Consortium (GICC) case–control study includes 103 cases (ages 18+) and 3287 controls

**Fig. 1:**
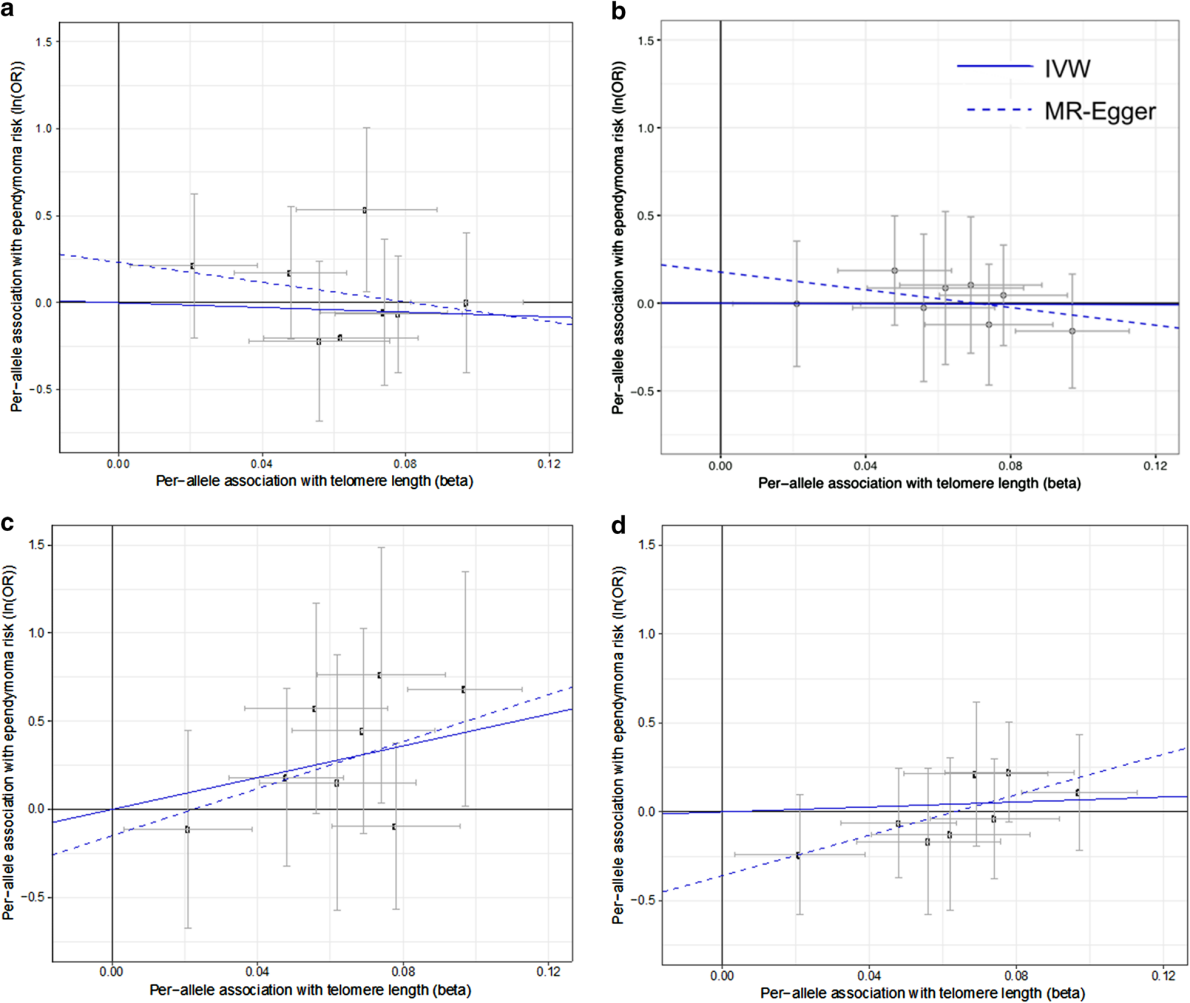
Per-allele association of ependymoma risk (y-axis) and leukocyte telomere length (x-axis) at eight SNPs known to influence telomere length, with the slopes of fitted lines equal to the inverse-variance weighted (IVW) Mendelian randomization estimate (solid line) and the MR-Egger estimate (dashed line) in **a** Toronto EPN-PF-A case–control data; **b** CCRLP childhood-onset (< 12 years) ependymoma case–control data; **c** CCRLP adolescent-onset (12–19 years) ependymoma case–control data; **d** Glioma International Case-Control Consortium (GICC) adult-onset ependymoma case–control data

In contrast to the CCRLP adolescent and the GICC adult ependymoma patients, no associations between LTL and ependymoma risk were observed for the CCRLP patients < 12 years of age or among the Toronto pediatric PF-A patients (median age, 3 years) in any of the MR estimates. We visualized the per-allele association of ependymoma risk (y-axis) plotted against the per-allele association with LTL (x-axis), with the slopes of the fitted lines equal to the IVW and MR Egger estimates (Fig. 1). The intercept is fixed at the origin for the IVW method and unconstrained for MR Egger. The slope is relatively flat for Toronto PF-A patients (Fig. 1a) and the CCRLP patients < 12 (Fig. 1b) using both IVW and MR Egger estimates, but is clearly positive in CCRLP patients age ≥ 12 for both IVW and MR Egger (Fig. 1c). The slope for the adult ependymoma analysis is relatively flat for the IVW estimate, but a significant positive slope was observed for the MR-Egger estimate (Fig. 1d).

## Discussion

Our results suggest that a genetic predisposition to longer telomere length increases the risk of adolescent and adult-onset ependymoma, but not risk of ependymoma diagnosed in children younger than 12 or in very young children with the EPN-PF-A molecular subtype. In addition to polygenic score analyses, the MR results in particular suggest that longer telomere length is a causal risk factor underlying adolescent ependymoma development. Although intracranial tumors are more common in children and spinal ependymomas are more common in adults [30], we observed that the association between genetic predisposition to longer LTL and adolescent-onset ependymoma risk was consistent in both the intracranial and spinal subgroups, as well as among the supratentorial tumors. Our findings contribute to the growing literature implicating longer telomere length as a risk factor for nervous system tumors in both children and adults [53, 55], and in a number of other non-nervous system cancers including lung, melanoma, chronic lymphocytic leukemia, and osteosarcoma [38, 52, 60].

Although the histopathologic distribution of primary CNS tumors differs in children and adults, childhood and adult-onset brain tumors likely share some common etiologic factors. Although adult-onset cancers are often linked to lifestyle and environmental risk factors while heritable and in utero exposures predominate in pediatric cancers, very few non-genetic risk factors have been identified for CNS malignancies [39]. While pediatric ependymomas have distinct genomic profiles from their adult counterparts, most notably in the epigenetically-driven EPN-PF-A subgroup that was not associated with telomere length in our analyses, telomerase reactivation has been observed to varying degrees across ependymoma subgroups and ages of onset [23, 30, 35].

We previously proposed that longer telomere length increases cancer risk by augmenting capacity for sustained cellular replication, allowing pre-malignant cells to accumulate the mutations necessary to resist apoptosis and enable replicative immortality [54], mutations such as hypermethylation of the *TERT* promoter [13, 23]. Our observation that genetic predisposition to longer LTL is associated with adolescent-onset and adult-onset ependymoma, but not childhood-onset ependymoma, supports this multi-step model of tumorigenesis in patients ages 12 and up. Longer telomere length may be an important mediator of ependymoma risk in adolescents and adults, where the tumor is more dependent upon acquiring somatic driver mutations and does not arise from an epigenetically dysregulated developmental cell lineage [26, 35].

In addition to polygenic score tests, we performed formal MR analyses to assess potential causal associations. These approaches are particularly useful for investigating the genetic epidemiology of pediatric malignancies given sample size limitations that hinder traditional GWAS approaches. Polygenic score analyses test genetic propensity for a phenotype, rather than a single variant, and have improved power over both single-SNP analyses and MR approaches [42, 43, 45]. Importantly, formal MR analyses can confirm the relationship between polygenic scores (longer LTL) and the outcome of interest (ependymoma) and provides a test of causal inference under valid assumptions that can be evaluated with various sensitivity analyses. MR analyses implied a causal relationship between longer telomere length and adolescent-onset ependymoma and there was no evidence of directional pleiotropy based on the null MR Egger intercept test. Our MR results lend support to a causal relationship between longer LTL and adolescent-onset ependymoma, complementing the polygenic score analyses.

In the analysis of GICC adult-onset ependymomas, longer LTL was positively associated with ependymoma risk in all MR models, although the IVW, weighted-median, and mode-based MR associations were not statistically significant. However, the MR Egger intercept test suggested that directional pleiotropy may be present. This directional pleiotropy, wherein genetic variants have pleiotropic effects that—on average—differ from zero, can result in biased IVW, weighted median, and mode-based estimates [7, 27]. Importantly, the MR Egger estimate, which is unbiased in the case of directional pleiotropy, suggested a positive causal association with LTL, consistent with the polygenic score and MR associations in adolescent-onset patients. Thus, the MR Egger estimate results suggest that genetic predisposition to longer LTL may be a risk factor for adult-onset ependymoma, although causal inference is complicated by the apparent presence of directional pleiotropy. Based on Fig. 1d, the primary source of this directional pleiotropy appears to be rs3027234 in *CTC1*. CTC1 is one of three members of the CST complex that binds to single-stranded DNA and is required to protect telomeres from DNA degradation. In addition to a role in telomere protection, the CST complex has a more general role in DNA metabolism at non-telomeric sites and was shown to protect DNA double-strand breaks from end resection, leading to repair by non-homologous end joining rather than homologous recombination [3].

Of note are the extreme magnitudes of the MR effect estimates in our analyses, which resulted in odds ratios (ORs) ranging from 91 to 368 per standard deviation of longer LTL score, with correspondingly large 95% confidence intervals. MR estimates are useful for testing causal relationships, but have limited utility in determining the exact size of a causal effect [47]. Our MR effect estimates may be inflated due to various reasons, such as age-specific variation in SNP-exposure or SNP-outcome associations, or under-estimation of genetic associations with the exposure compared to the outcome. Importantly, the large ORs observed in our analysis could also be a result of unstable estimates due to small sample sizes. However, a previous comprehensive MR study across multiple cancer types suggests that larger MR associations tend to be seen for tissue sites with lower rates of stem cell division, such as the brain, with the largest such estimate observed for glioma (OR, 5.27). Thus, we do not rule out the possibility that longer LTL may have a large magnitude of effect on ependymoma risk in adolescents and adults.

The role of telomere maintenance has been extensively investigated in ependymoma, although our study appears to be the first to investigate germline modifiers of telomere length in ependymoma etiology. The telomerase enzyme is normally expressed in stem and progenitor cells to maintain telomere length, but is suppressed in somatic tissues. Telomerase activity is reactivated in many cancer subtypes, including ependymomas [4, 9, 13, 23, 31]. Telomerase activity has been linked to ependymoma progression, recurrence, and survival, and has been implicated as an important prognostic marker and therapeutic target [23, 50, 51], where telomerase inhibition has demonstrated anti-tumorigenic effects in in vitro and xenograft models of pediatric ependymoma [4, 58]. Telomere dysfunction has also been linked to chromothrypsis, a form of genomic instability characterized by tens to hundreds of clustered DNA rearrangements, which was previously associated with greater telomere length in medulloblastoma and ependymoma [20]. *TERT* promotor mutations that reactivate telomerase in glioblastoma have occasionally been identified in adult ependymoma, but not in children [4, 9, 31]. In pediatric ependymoma, hypermethylation of the *TERT* promotor has consistently been associated with telomerase reactivation [13, 23], indicating that epigenetic mechanisms of telomere maintenance may also enable replicative immortality in ependymoma cells. Germline variants, including methylation quantitative trait loci (meQTLs), may accelerate or even enable such epigenetic reactivation. Our results build upon this body of literature by demonstrating that constitutively longer telomere length/inherently better telomere maintenance is associated with ependymoma predisposition in adolescents and adults.

A strength of this study is that our age-stratified findings in the CCRLP dataset were supported by independent datasets of molecularly-subgrouped pediatric cases (Toronto) and adult-onset cases (GICC). However, our study has limitations. Ependymoma is a rare malignancy, so our study is limited by its relatively modest sample size. Despite being better powered to observe a significant association in the childhood-onset ependymoma cases, we still detected a significant association in the smaller subset of adolescent-onset ependymoma cases. There are also limitations to our MR analyses, including the issue of horizontal pleiotropy, discussed earlier, and potential violations of MR assumptions. The assumptions include: (1) a consistent log-linear association between telomere length and cancer risk; (2) that LTL-associated variants have similar associations in ependymal cells; and (3) that the LTL variants derived from an adult population have similar associations in children and adolescents. Violations of any of these assumptions would likely result in a bias toward the null, so the significant associations observed in our data are more likely to be attenuated than to be inflated. However, we cannot rule out the possibility that LTL variants used in this study are less associated in a pediatric population [49], resulting in a null association among the Toronto EPN-PF-A and CCRLP < 12 subsets.

In summary, we leverage a polygenic score and MR framework to examine whether longer telomere length may be a risk factor for ependymoma across age strata. Our findings indicate that genetic predisposition to longer LTL is associated with increased risk of adolescent- and adult-onset ependymoma, but not with childhood ependymoma, including EPN-PF-A. These findings complement emerging literature suggesting that dysregulated telomere maintenance is important for ependymoma pathogenesis and that longer telomere length is a risk factor for several different nervous system malignancies. Future studies should work to incorporate germline data into genomic and epigenomic profiling of ependymoma tumors to explore the relationship between heritable variation and telomerase activity in somatic cells.

## Supplementary information

**Additional file 1:** **Supplementary note:** The Glioma International Case Control Study membership. **Supplementary Table 1.** Demographic and clinical data for pediatric ependymoma cases (ages 0-19) from the California Cancer Record Linkage Project (CCRLP). **Supplementary Table 2.** Single SNP associations between LTL-associated variants and ependymoma risk in the CCRLP case-control dataset, stratified by age at diagnosis. **Supplementary Table 3.** Single SNP associations between LTL-associated variants and ependymoma risk in the Toronto and GICC case-control datasets. **Supplementary Table 4.** Association between polygenic score for longer leukocyte telomere length and risk of ependymoma in the California Cancer Records Linkage Project (CCRLP)^a^, stratified by tumor site (spinal vs. intracranial) and age (<12, ≥12). Associations are adjusted for sex and first 10 principal components

## Data Availability

This study used biospecimens from the California Biobank Program. Any uploading of genomic data and/or sharing of these biospecimens or individual data derived from these biospecimens has been determined to violate the statutory scheme of the California Health and Safety Code Sections 124980(j), 124991(b), (g), (h), and 103850 (a) and (d), which protect the confidential nature of biospecimens and individual data derived from biospecimens. Certain aggregate results may be available from the authors by request.
