# Supplementary material for: Genetic predisposition to longer telomere length and risk of childhood, adolescent and adult-onset ependymoma
Source: Acta Neuropathol Commun. 2020 Oct 28;8:173. doi: 10.1186/s40478-020-01038-w (PMC7592366; doi:10.1186/s40478-020-01038-w)
Supplement: Supplementary file 1 — Additional file 1: Supplementary note: The Glioma International Case Control Study membership. Supplementary Table 1. Demographic and clinical data for pediatric ependymoma cases (ages 0-19) from the California Cancer Record Linkage Project (CCRLP). Supplementary Table 2. Single SNP associations between LTL-associated variants and ependymoma risk in the CCRLP case-control dataset, stratified by age at diagnosis. Supplementary Table 3. Single SNP associations between LTL-associated variants and ependymoma risk in the Toronto and GICC case-control datasets. Supplementary Table 4. Association between polygenic score for longer leukocyte telomere length and risk of ependymoma in the California Cancer Records Linkage Project (CCRLP)a, stratified by tumor site (spinal vs. intracranial) and age (<12, ≥12). Associations are adjusted for sex and first 10 principal components [file 40478_2020_1038_MOESM1_ESM.docx]

**Supplementary note:**

The Glioma International Case Control Study membership currently includes: Elizabeth B. Claus (School of Public Health, Yale University, New Haven, CT 06510, USA and Department of Neurosurgery, Brigham and Women’s Hospital, Boston, MA 02115, USA); Dora Il’yasova (Department of Epidemiology and Biostatistics, School of Public Health, Georgia State University, Atlanta, GA 30303, USA; Duke Cancer Institute, Duke University Medical Center, Durham, NC 27710, USA and Cancer Control and Prevention Program, Department of Community and Family Medicine, Duke University Medical Center, Durham, NC 27710, USA); Kyle M. Walsh (Department of Neurosurgery and Preston Robert Tisch Brain Tumor Center, Duke University Medical Center, Durham, NC, 27710, USA); Joellen Schildkraut (Department of Public Health Sciences, School of Medicine, University of Virginia, Charlottesville, VA 22903, USA); Jill S. Barnholtz-Sloan (Department of Population and Quantitative Health Sciences and the Cleveland Center for Health Outcomes Research, Case Western Reserve University School of Medicine, Cleveland, OH 44106, USA); Sara H. Olson (Department of Epidemiology and Biostatistics, Memorial Sloan Kettering Cancer Center, New York, NY 10017, USA); Jonine L. Bernstein (Department of Epidemiology and Biostatistics, Memorial Sloan Kettering Cancer Center, New York, NY 10017, USA); Christoffer Johansen (Danish Cancer Society Research Center, Survivorship, Danish Cancer Society, Copenhagen 2100, Denmark; 15Oncology Clinic, Finsen Centre, Rigshospitalet, University of Copenhagen, Copenhagen 2100, Denmark); Robert B. Jenkins (Department of Laboratory Medicine and Pathology, Mayo Clinic Comprehensive Cancer Center, Mayo Clinic, Rochester, MN 55905, USA); Beatrice S. Melin (Department of Radiation Sciences, Umeå University, Umeå 901 87, Sweden); Margaret R. Wrensch (Department of Neurological Surgery, School of Medicine, University of California, San Francisco, CA 94143, USA); Richard S. Houlston (Division of Molecular Pathology, The Institute of Cancer Research, London SW7 3RP, UK); Melissa L. Bondy (Department of Epidemiology and Population Health, Stanford Cancer Institute, Stanford University, Stanford, CA 94305, USA)

**Table S1.** Demographic and clinical data for pediatric ependymoma cases (ages 0-19) from the California Cancer Record Linkage Project (CCRLP)

| Demographic and/or clinical variable^a^ | Mean (SD) or n (%) |
| --- | --- |
| Age at diagnosis | 6.7 (6.0) |
| ≥ 12 years | 39 (21.3%) |
| Sex, male | 81 (52.9%) |
| Spinal tumor^b^ | 26 (17.0%) |
| Histology |  |
| Anaplastic Ependymoma | 36 (23.5%) |
| Myxopapillary | 14 (9.2%) |
| Sub-ependymoma | 2 (1.3%) |
| Papillary | 1 (0.6%) |
| Ependymoma (Not otherwise specified) | 100 (65.4%) |
| Grade |  |
| I | 8 (5.2%) |
| II | 16 (10.5%) |
| III | 9 (5.9%) |
| IV | 32 (20.9%) |
| Not otherwise specified | 88 (57.5%) |

^a^ n = 153 self-reported non-Hispanic white ependymoma patients

^b^ Alternative tumor site/location is intracranial

**Table S2.** Single SNP associations between LTL-associated variants and ependymoma risk in the CCRLP case-control dataset, stratified by age at diagnosis

|  |  | Age <12 years^1^ | | Age ≥12 years^2^ | |
| --- | --- | --- | --- | --- | --- |
| SNP | **Gene** | **OR (95% CI)** | **P-value** | **OR (95% CI)** | **P-value** |
| rs11125529 | *ACYP2* | 0.97 (0.63, 1.46) | 0.901 | 1.77 (0.95, 3.16) | 0.059 |
| rs10936599 | *TERC* | 0.85 (0.62, 1.19) | 0.337 | 1.98 (1.06, 4.06) | **0.043** |
| rs7675998 | *NAF1* | 0.88 (0.63, 1.26) | 0.486 | 2.15 (1.09, 4.74) | **0.039** |
| rs2736100 | *TERT* | 1.05 (0.79, 1.39) | 0.759 | 0.91 (0.57, 1.46) | 0.69 |
| rs9420907 | *OBFC1* | 1.11 (0.74, 1.62) | 0.603 | 1.56 (0.85, 2.74) | 0.13 |
| rs3027234 | *CTC1* | 1.00 (0.70, 1.44) | 0.984 | 0.89 (0.52, 1.61) | 0.70 |
| rs8105767 | *ZNF208* | 1.20 (0.88, 1.64) | 0.243 | 1.20 (0.72, 1.97) | 0.48 |
| rs755017 | *RTEL1* | 1.09 (0.69, 1.66) | 0.698 | 1.16 (0.54, 2.31) | 0.68 |

^1^n=114 cases, n=696 controls

^2^n=39 cases, n=696 controls

P values < 0.05 in bold. Odds ratio (OR) for risk of ependymoma associated with allele that increases telomere length.

**Table S3.** Single SNP associations between LTL-associated variants and ependymoma risk in the Toronto and GICC case-control datasets

|  |  | Toronto (n=83 cases, n=332 controls) | | GICC (n=103 cases, n=3287 controls) | |
| --- | --- | --- | --- | --- | --- |
| SNP | **Gene** | **OR (95%CI)** | **P-value** | **OR (95%CI)** | **P-value** |
| rs11125529 | *ACYP2* | 0.80 (0.51, 1.27) | 0.347 | 0.85 (0.56, 1.28) | 0.43 |
| rs10936599 | *TERC* | 1.00 (0.67, 1.50) | 1.000 | 1.12 (0.81, 1.54) | 0.50 |
| rs7675998 | *NAF1* | 0.95 (0.62, 1.44) | 0.798 | 0.96 (0.69, 1.35) | 0.83 |
| rs2736100 | *TERT* | 0.94 (0.67, 1.31) | 0.706 | 1.25 (0.95, 1.65) | 0.13 |
| rs9420907 | *OBFC1* | 1.71 (1.07, 2.73) | **0.026** | 1.24 (0.83, 1.85) | 0.30 |
| rs3027234 | *CTC1* | 1.24 (0.82, 1.87) | 0.311 | 0.79 (0.56, 1.10) | 0.16 |
| rs8105767 | *ZNF208* | 1.19 (0.81, 1.74) | 0.373 | 0.94 (0.69, 1.28) | 0.69 |
| rs755017 | *RTEL1* | 0.82 (0.46, 1.44) | 0.488 | 0.88 (0.57, 1.35) | 0.56 |

^1^ Odds ratio (OR) for risk of ependymoma associated with allele that increases telomere length

^2^ 95% confidence interval

Nominally significant P values < 0.05 in bold.

**Table S4.** Association between polygenic score for longer leukocyte telomere length and risk of ependymoma in the California Cancer Records Linkage Project (CCRLP)^a^, stratified by tumor site (spinal vs. intracranial) and age (<12, ≥12). Associations are adjusted for sex and first 10 principal components.

| Subtype, age | Cases/controls ^a^ | OR (95% CI) | P-value |
| --- | --- | --- | --- |
| Spinal, all | 26/696 | 1.09 (0.74-1.62) | 0.66 |
| Spinal, <12 | 8/696 | 0.33 (0.13-0.72) | **8.9x10^-3^** |
| Spinal, ≥12 | 18/696 | 1.79 (1.02-2.12) | **0.024** |
| Intracranial, all | 122/696 | 1.14 (0.93-1.39) | 0.47 |
| Intracranial, <12 | 104/696 | 1.07 (0.87-1.33) | 0.53 |
| Intracranial, ≥12 | 18/696 | 1.66 (1.01-2.77) | **0.048** |
| Supratentorial, all | 33/696 | 1.32 (0.98-1.07) | 0.31 |
| Supratentorial, <12 | 25/696 | 1.00 (0.95-1.05) | 0.86 |
| Supratentorial, ≥12 | 8/696 | 1.72 (1.01-1.23) | **0.024** |

^a^n = 148 self-reported non-Hispanic white ependymoma patients from the California Cancer Records Linkage Project and 696 controls. Five cases lacked tumor site data.

P values <0.05 in bold.
